# Selected meeting abstracts of 2nd International Conference on Contemporary Oncology

**DOI:** 10.37349/etat.2024.00247

**Published:** 2024-06-28

**Authors:** Chouaib Salem

**Affiliations:** INSERM UMR 1186, Integrative Tumour Immunology and Immunotherapy, Gustave Roussy, Fac. de Médecine - Univ. Paris-Sud, Université Paris-Saclay, 94805, Villejuif, France

## Introduction:

Rhabdomyosarcoma is the most common type of soft tissue sarcoma in infants. It originates from the embryonic mesenchymal cell, which is the embryonic support tissue that from the basis of various tissues in adults. It can, therefore, have various locations in the body. The perineal region is, however, very rare.

## Case report:

We report a case of a 16-year-old girl who presented with a mass in the right upper vaginal lip, treated twice with antibiotics for suspected bartholinitis. On examination, a 3 cm mass, painless, taking the whole right lip, with continuity to the peri-anal region, without associated adenopathies. On pelvic MRI, the mass was perineal and infiltrated the puborectal muscle and the tendinous center of the perineum. Thus, in close contact with the external anal sphincter. Biopsy was performed for histological and immunohistochemical study. The results were in favour of a perineal embryonic RMS expressing desmin and myogenin.

## Conclusions:

Early diagnosis of perineal rhabdomyosarcoma in adolescents and children is crucial. Management during a multidisciplinary meeting is one of the challenges facing oncologists and surgeons. One of these challenges is the preservation of fertility. The study was approved by local ethic committee of gynecology and obstetrics department.

