# Supplementary material for: Selected meeting abstracts of 2nd International Conference on Contemporary Oncology
Source: Explor Target Antitumor Ther. 2024 Jun 28;5(3):789–99. doi: 10.37349/etat.2024.00247 (PMC11220287; doi:10.37349/etat.2024.00247)
Supplement: Related texts

# Abstract: 02 Distribution of TRAF-6 and its relationship to the proliferation marker Ki-67 in prostate cancer

**Published:** 2024-06

**Authors:** Awatef Ben Jemaa, Hanen Kahlaoui, Oumaima Khélifi, Sataa Sallami, Yassine Nouira, Ahmed Banasr, Ridha Oueslati

**Affiliations:** 1 Unit of Immunology and Microbiology Environmental and Carcinogenesis (IMEC), Faculty of Sciences of Bizerte, University of Carthage, Bizerte 7021, Tunisia; 2 Department of Biology, Faculty of Science of Gafsa, University of Gafsa, Gafsa 2112, Tunisia; 3 Department of Urology, Hospital of La Rabta, Tunis 1007, Tunisia; 4 Department of Legal Medicine, Hospital of Charles Nicolle, Tunis 1006, Tunisia

## Aim:

TRAF-6 factor is an adapter protein that mediates a wide range of protein-protein interactions. Although TRAF-6 has been shown to have oncogenic activity in various malignancies, its exact role in prostate carcinogenesis remains poorly elucidated. In order to decide on the pro- or anti-tumor role of TRAF-6 in the prostate tumor, our study on this immunological marker was carried out in parallel with the proliferation marker Ki-67.

## Methods:

The study was carried out in 4 normal (NP), 5 benign prostatic hyperplastic (BPH) and 16 cancerous human prostates (PC). Immunohistochemical and analysis was performed. Serum levels of PSA were assayed by an immulite autoanalyzer.

## Results:

Considering the intensity of immunostaining, TRAF-6, and Ki-67 were upregulated in PC samples compared to NP and BPH. Moreover, TRAF-6 was inversely related to sera PSA in PC patients. Interestingly, the activation status of TRAF-6 and Ki-67 varies between prostate groups and between the stromal and glandular component. In cancer patients, TRAF-6 and Ki-67 are co-expressed in the nucleus of stromal cells. In contrast, in neoplastic cells, TRAF-6 accumulated in the cytoplasm while Ki-67 occupied a nuclear position in most cancer patients. In PN, the TRAF-6/Ki-67 couple is completely absent. In benign patients, the TRAF-6(-)/Ki-67(-) profile is predominant in endothelial cells. In contrast, co-expression of TRAF-6 and Ki-67 was revealed in endothelial cells only in cancer patients.

## Conclusions:

Taken together, our study argues for a pro-tumor role of TRAF-6 in PC. Our results validate the interest of the TRAF-6/Ki-67 coupling, the status of the two markers of which was synergistic in the PC. This extraordinary synergy between TRAF-6 and Ki-67 status should be considered to better understand anti-tumor immunity in prostate tumors. This study was approved by the Ethics Commission of La Rabta Hospital (61/2020). Informed consent to participate in the study was obtained from all participants. Informed consent to publication was obtained from relevant participants.

# Abstract: 03 Mutation of isocitrate dehydrogenase in glioblastoma: exploration of a new predictive tool

**Published:** 2024-06

**Authors:** Ghammem Nour, Ayari Jihen, Ben Naser Sonia, Balti Mehdi, Haddaoui Abderrazek

**Affiliations:** Medical Oncology Department, The Military hospital of Tunis, Tunis 1008, Tunisia

## Aim:

Despite advancements in glioblastoma treatment, its prognosis remains fatal with significant heterogeneity. This study examines the epidemiological profile, treatment, and prognosis of glioblastoma, focusing on the IDH1-R132H mutation’s distribution and diagnostic, therapeutic, and prognostic value.

## Methods:

A descriptive and statistical analysis was conducted on 45 patients from January 2012 to December 2020 at the Medical Oncology Department of The Military hospital of Tunis. The study compared the survival rates of IDH1-R132H mutated glioblastomas with those of IDH-wild glioblastomas and examined potential associated prognostic factors.

## Results:

The results revealed a male-predominant cohort (77%), aged 18 years to 76 years (mean age: 53). Glioblastoma symptoms included intracranial hypertension (42%), motor deficits (60%), and higher function disorders (24%). Computed tomography was initially requested for 43 patients, with MRI for only two. The most common location was the frontal lobe. Surgical procedures involved total (51%), subtotal (14%), or partial removal (34%). Postoperative treatment was received by 30 patients, with 14 undergoing radiotherapy and 16 undergoing radiotherapy combined with chemotherapy. The IDH1-R132H mutation was found in 18 patients (40%), more prevalent in secondary glioblastoma (97%), and associated with younger age and better functional scores. Overall survival was 12.4 months, with IDH-mutated glioblastomas showing longer survival (16.8 months) compared to IDH-wild-type (9.5 months). Progression-free survival was also better in the IDH-mutated group (12.9 months vs. 4.5 months). Cox model analysis of other prognostic factors indicated superior survival with a better preoperative Kaofsky functional score (*P* = 0.005), complete resection versus incomplete resection (*P* = 0.005; 15.6 months and 8.6 months, respectively, *P* = 0.025), and adjuvant radio-chemotherapy (*P* = 0.016).

## Conclusions:

In conclusion, surgical extent, Kaofsky index, and adjuvant radiotherapy or radiochemotherapy impact glioblastoma prognosis. The IDH1-R132H mutation identifies a subgroup with a better prognosis, warranting routine use after recent advancements in immunohistochemistry analysis. The study is exempt from ethical approval. Informed consent to participate in the study was obtained from all participants. Informed consent to publication was obtained from relevant participants.

# Abstract: 04 Breast cancer in its family form: diagnostic strategy and therapeutic management

**Published:** 2024-06

**Authors:** Haifa Rachdi, Yosra Bouzguenda, Myriam Saadi, Nesrine Mejri, Nouha Daoud, Hamouda Boussen

**Affiliations:** Medical Oncology Department, Abdurrahman Mami Hospital, Ariana, Tunisia

## Aim:

Family breast cancer is present in 5–10% of cases in a family context where mutations of the *BRCA1* and *BRCA2* genes are mainly incriminated. In Tunisia, several families are known to carry these mutations. The objective of our work was to describe the anamnestic, clinical, radiological, and biogenetic characteristics of this form of cancer, and to determine its therapeutic particularities.

## Methods:

We conducted a retrospective analysis of patients treated for breast cancer with genetic mutation between January 2011 and May 2021 in the Medical Oncology Department of Abdurrahman Mami Hospital, Ariana.

## Results:

BRCA1 mutation was found in 14 cases (50%), BRCA2 in 11 cases (39.3%), CHEK2 in 2 cases, and TP53 in 1 case. Twenty-four patients had a family history of breast cancer. A majority of 85.7% had an indication at an oncogenetic consultation based on the Eisinger score. The other most common neoplasia was ovarian tumours in 16 cases, digestive tumours in 10 cases, and lung cancer in 8 cases. Of our patients, 72.1% aged less than 40 years had the BRCA1 mutation, while 58.8% of patients aged 40 years or older had the BRCA2 mutation. Among patients with the BRCA1 mutation 81.8% were triple-negative and 85.7% of patients with the BRCA2 mutation were luminal B. There was an indication for treatment with PARP inhibitors in 5 patients, but none of them received this treatment because of its unavailability in our country.

## Conclusions:

This work is the first one developed in Tunisia that addresses the subject of breast cancer by approaching the clinical component associated with genetic analysis results. Epidemiological and anamnestic results show the usefulness of genetic screening and preventive measures in this type of cancer. Therapeutic outcomes justify the need to invest in targeted cancer therapies to improve the management of hereditary breast cancer. This study exemption from ethical approval. Written informed consent was obtained from the patient for publication of this case report and accompanying images.

# Abstract: 05 Concomitant radiotherapy and cyclin-dependent kinase 4/6 inhibitor for metastatic breast cancer: a risky or a beneficial combination?

**Published:** 2024-06

**Authors:** Kchaou Lina, Ben Rejeb Mouna, Rachdi Haifa, Moujahed Rim, Ghorbal Lilia, Hamdoun Watef, Kochbati Lotfi

**Affiliations:** 1 Radiotherapy Oncology Department, Abderrahman Mammi Hospital, Ariana, Tunisia; 2 Medical Oncology Department, Abderrahman Mammi Hospital, Ariana, Tunisia

## Aim:

Given the well-established role of CDK4/6 inhibitors (CDK4/6inh) in hormone receptor positive, human epidermal growth factor receptor 2 negative metastatic breast cancer (MBC), the incidence of patients requiring radiotherapy (RT) is likely to increase. This study aims to assess the acute toxicity of concomitant RT and CDK4/6inh for patients treated in our institution.

## Methods:

We conducted a retrospective study of MBC patients on CDK4/6inh who underwent concurrent RT. We reviewed toxicity in the form of skin changes, neutropenia, gastrointestinal manifestations. Toxicity was assessed according to the National Cancer Institute Common Terminology Criteria for Adverse Events v5. Laboratory tests performed up to 1 month after radiation treatment were reviewed to assess hematologic toxicities.

## Results:

Of the 20 patients included in the study, 13 received ribociclib (65%) and 7 palbociclib. The median age was 49.5 (35–70) at the time of RT. Radiotherapy included 135 multifraction courses and 10 single fraction courses. The median total dose was 26 Gy (6–40 Gy). The majority of patients (55%) received breast (local/regional) irradiation following either hypofractionated regimen (40 Gy/15 fractions) or ultra-hypofractionated regimen (26 Gy/5 fractions). Nine patients underwent palliative RT treatment as follows: spinal metastases (*n* = 7), long bones (*n* = 1), pelvis (*n* = 1). Patients were treated using 3DCRT technique. The most common toxicity was hematological toxicity; grade 2 neutropenia was observed in 30% of cases. No thrombocytopenia was assessed. One patient had grade 2 skin ulceration. One patient experience grade 1 dysphagia. No other acute toxicities were observed. No patients had to stop CDK4/6inh or RT due to toxicity and no patients required dose reduction of CDK4/6inh during or after RT.

## Conclusions:

Based on our experience, the concomitant treatment of CDK4/6inh and RT seems to be well tolerated, with no enhanced toxicities. Larger studies are needed to elucidate the safety of this combination. The study was exempted from ethical approval.

# Abstract: 06 Clinical-dosimetric relationship between lacrimal gland dose and ocular toxicity after intensity-modulated radiotherapy for nasopharyngeal carcinoma

**Published:** 2024-06

**Authors:** Kchaou Lina, Zarraa Semia, Yousfi Amani, Noubbigh Ghaiet El Fida, Yahiaoui Safia, Nasr Chiraz

**Affiliations:** 1 Radiotherapy Oncology Department, Salah Azaiez Institute, Tunis, Tunisia; 2 Radiotherapy Oncology Department, The Military Hospital, Tunis, Tunisia

## Aim:

To evaluate the relationship between lacrimal gland dose and ocular toxicity among the patients treated by intensity modulated radiotherapy (IMRT) for nasopharyngeal carcinoma (NPC).

## Methods:

Between September 2020 and May 2022, we selected 20 patients treated for NPC by IMRT technique to a curative dose of 70 Gy at Salah Azaeiz Institute. The lacrimal gland was contoured on the axial slice as organ at risk (OAR). The following dosimetric parameters were evaluated: mean dose and maximum dose. A tear break-up time (TBUT) test was conducted to assess dry eye syndrome.

## Results:

The median follow-up time was 9 months (5–22 months). Based on the dose-volume histogram analysis, averages of mean and maximum doses to the ipsilateral lacrimal gland were 11.4 Gy (range 3.7–29.9 Gy) and 30.3 Gy (range 14.4–46.9 Gy), respectively. The median time of the TBUT test was 7 s. The incidence of late grade 3 + toxicities was 15%. We reported a severe dry eye syndrome in two patients and the mean dose to their lacrimal gland were superior to 25 Gy.

## Conclusions:

High-grade ocular toxicity is a potential complication that detrimentally affects the quality of life in patients treated by IMRT for NPC. The incidence of these complications has been shown to increase with higher doses to the lacrimal gland. To reduce the toxicity and prevent dry eye syndrome lacrimal gland should be contoured as OAR and dose constraints should be given. The project registered under reference ISA/2023/17 obtained a favorable opinion of Salah Azaiez Ethics Committee. Written informed consent was obtained from the patients.

# Abstract: 07 Radiation oncology outpatient satisfaction with cancer care: the quality of care from patient’s perspective

**Published:** 2024-06

**Authors:** Zeineb Naimi, Meriem ElBessi, Meriem Bohli, Raouia Ben Amor, Awatef Hamdoun, Ghada Bouguerra, Rihab Haddad, Lotfi Kochbati

**Affiliations:** Radiation Oncology Department, Abderrahmen Mami Hospital Ariana, Ariana 2080, Tunisia

## Aim:

The aim of this study was to evaluate patient’s satisfaction with cancer care in the setting of outpatient radiotherapy.

## Methods:

We conducted a cross-sectional study including all patients undergoing radiotherapy during April 2023. Patients were treated with either 3D conformal radiotherapy (3DCRT) or volumetric modulated arc therapy (VMAT). They were asked to fill in the EORTC OUT-PATSAT35 RT questionnaire. Patients coming for their first session were excluded. Domain’s scores were calculated and statistically analyzed.

## Results:

A total of 70 patients were included in this study. Forty-seven (67%) patients were treated with 3DCRT and 23 (33%) with VMAT. Mean age was 56 (12–98) years old. Sixty-nine percent of the patients were female. The mean overall satisfaction (OS) score was 84.3. All domains’ scores statistically correlated with the OS mean scores in the different domains regarding doctors, radiation therapists and services/organization are reported in Table 1. A “poor” cotation was reported in 11.6% of cases in item n° 32 (accessibility), 6% of the cases in item n° 29 (waiting time) and in 3% of the cases in items n° 28 and 33 (information about other services and ease in finding the different units). Fourteen patients didn’t answer item n° 25 (exchange of information with services outside of the hospital). Eight patients added comments to the questionnaire (acknowledgments in seven cases and a recommendation in one case).

**Table 1:** Means scores of the EORTC OUT-PATSAT35 RT questionnaire

**Domain**	**Mean score**
Doctors	
Technical skills	81.8 (33.3–100)
Interpersonal skills	81.3 (41.7–100)
Information provision	75.1 (8.33–100)
Availability	79.5 (25–100)
Radiation therapists	
Technical skills	87.0 (37.5–100)
Interpersonal skills	86.2 (50–100)
Interpersonal skills	77.0 (25–100)
Availability	79.6 (25–100)
Services/Organization	
Exchange of information	77.2 (25–100)
Information provided	79.3 (16.7–100)
Waiting time	70.2 (16.7–100)
Environment	71.1 (25–100)

## Conclusions:

We found globally high satisfaction scores with cancer care in our department. The lowest scores were mainly in the organization domain, suggesting that an extra effort should be made to improve radiotherapy outpatient experience. The EORTC OUT-PATSAT35 RT seems to be a reliable and valid instrument to identify which aspect of care should be improved. The study was approved by the Ethics Committee of Abderrahmen Mami Hospital Ariana. Informed consent to participate in the study was obtained from all participants.

# Abstract: 08 Mediastinal irradiation for thymic epithelial tumors: a deep focus on coronary arteries’ exposure

**Published:** 2024-06

**Authors:** Zeineb Naimi, Meriem ElBessi, Meriem Bohli, Raouia Ben Amor, Awatef Hamdoun, Ghada Bouguerra, Rihab Haddad, Lotfi Kochbati

**Affiliations:** Radiation Oncology Department, Abderrahmen Mami Hospital Ariana, Ariana 2080, Tunisia

## Aim:

This study aimed to assess coronary arteries exposure in mediastinal radiotherapy for thymic tumors.

## Methods:

All thymic tumors patients referred for radiotherapy, between 2017 and 2022, were included in this study. Patients were treated with 3D conformal free breathing radiotherapy. Cardiac subvolumes and coronary arteries, including the left main (LM), left anterior descending (LAD), left circumflex (Cx) and the right coronary artery (RCA), were contoured using the Duane’s cardiac atlas. Dose volume histograms were generated for all structures. Radiation exposure to coronary arteries was assessed and analysed with regards to the prescription dose, target volumes and usual dose constraints to the heart.

## Results:

A total of 22 patients with thymic tumors were included. Mean age at diagnosis was 51 years, ranging between 23 years and 70 years. Patient’s distribution according to Masaoka-Koga/ITMIG tumor stage was as follow: 72% of stage IIB, 16.7% of stage III, and 11.3% of stage IV disease. Thymoma was the main histological subtype (90% of cases) with type B2, B2/B3 and B3, respectively in 44.5%, 11% and 5.6% of cases. Two patients were treated for thymic carcinoma. Fifteen patients (83.3%) underwent surgery. The mean radiation prescription dose was 54.5 Gy, ranging between 45 Gy and 60 Gy. Usual dose constraints to the heart were achieved for all patients. The mean Dmean/Dmax for coronary arteries were as follow: 35.6 Gy/40.4 Gy for LM, 21.39 Gy/38.5 Gy for LAD, 14.73 Gy/39 Gy for Cx, and 12.61 Gy/32.04 Gy for RCA. Strong positive correlation was found between usual heart dose constraints (Dmean heart/V30 Gy) and doses delivered to coronary arteries, with Pearson coefficient ranging between 0.57 and 0.82. Exclusive irradiation radiotherapy yielded higher point doses to proximal coronary arteries’ segments when compared to post-operative radiotherapy (*P* = 0.03).

## Conclusions:

Mediastinal radiotherapy for thymic tumors yielded substantially high doses to all coronary arteries (Dmax > 30 Gy). High point doses were reported to coronary arteries’ proximal segments, suggesting a high risk of potentially harmful radiation induced coronary disease. Heart sparing radiotherapy techniques should be routinely used in thymic tumors irradiation. The study was approved by the Ethics Committee of Abderrahmen Mami Hospital Ariana. Informed consent to participate in the study was obtained from all participants.

# Abstract: 09 Therapeutic and prognostic aspects of ovarian cancer: the experience of the Main Instructional Military Hospital (MIMH) in Tunis

**Published:** 2024-06

**Authors:** Ghammem Nour, Ayari Jihen, Ben Naser Sonia, Balti Mehdi, Haddaoui Abderrazek

**Affiliations:** Department of Medical Oncology, The Military Hospital of Tunis, Tunis 1008, Tunisia

## Aim:

Ovarian cancer (OC) ranks fifth among cancers and fourth as a leading cause of cancer-related deaths in women. This study aims to update the therapeutic and prognostic data on OC in Tunisia.

## Methods:

A retrospective observational study was conducted at MIMH’s Medical Oncology Department, using records of primary OC patients receiving treatment between 2010 and 2016. Descriptive and analytical univariate analyses (Kaplan-Meier method and log rank test at *P* < 0.05) and multivariate analyses (linear regression at *P* < 0.15) were performed.

## Results:

Thirty OC cases were analyzed. The time from diagnosis to treatment initiation averaged 28 days (range: 3–48 days). Radical surgery was done early in seven cases, while 11 had incomplete surgery. First-line CT was indicated for all patients, administered adjuvantly in 73.3%, mainly at early stages (77.8%), with an average of 5 cycles. Carboplatin and paclitaxel-based regimens were given to 96.7% with average doses of 266 mg (range: 130–560 mg) and 523 mg (range: 250–500 mg), respectively. CT-related toxicity affected 26 patients, most commonly fatigue (20 cases), followed by gastrointestinal (12 cases), hematologic (11 cases), and neurological (10 cases) toxicities. Eighteen out of 26 cases achieved complete remission, and CA125 normalized in 12 out of 15 patients. Recurrence occurred in four patients, with two local and two distant cases. The mean time to the first recurrence was 28 months (range: 3–48 months). Average progression-free survival was 8.5 months (range: 0–51 months), and overall survival was 63.3 months (range: 39.6–87 months). Follow-up was limited; only 56.7% of patients were followed over a period of 1 year and 13.3% over a period of 3 years. No statistically significant predictive factors for treatment response were identified.

## Conclusions:

Despite therapeutic advancements in Tunisia, OC still faces high recurrence and mortality rates. Targeted therapies like bevacizumab and PARP inhibitors show promise for improving the prognosis. The study is exempt from ethical approval. Informed consent to participate in the study was obtained from all participants. Informed consent to publication was obtained from relevant participants.

# Abstract: 10 Coincidental splenic irradiation in lower lobe lung tumors treatment: should we worry?

**Published:** 2024-06

**Authors:** Inès Mlayah, Mouna Ben Rejeb, Rim Moujahed, Lilia Ghorbel, Awatef Hamdoun, Rihab Haddad, Ghada Bouguerra, Lotfi Kochbati

**Affiliations:** Radiotherapy Oncology Department, Abderrahmane Mami Hospital, Ariana, Tunis 2080, Tunisia

## Aim:

Recent data have shown that lymphopenia, a common complication of radiotherapy, has a detrimental impact on survival. Studies have also suggested that there is a correlation between the dose received by the spleen and the risk of lymphopenia. However, there are no validated dosimetric constraints for the spleen. The purpose of this study was to evaluate the dose received by the spleen during external beam radiotherapy of lower lobe lung tumors and to investigate the association between the delivered dose to the spleen and the risk of lymphopenia.

## Methods:

A retrospective analysis of the medical records of 15 patients undergoing sequential chemo-radiotherapy between 2018 and 2022 for lower lobe lung cancer was performed. The mean dose prescribed to the planning target volume (PTV) was 57.8 Gy (45–64 Gy). The spleen was not considered as an organ at risk (OAR) during treatment planning and was delineated retrospectively. Complete blood counts were done and collected before and after the end of radiotherapy (RT). The delta lymphocyte (delta lymph), corresponding to the variation in the absolute number of lymphocytes before and after the completion of radiotherapy, was calculated. The dosimetric variables analyzed were the mean dose (Dmean), the maximum dose (Dmax), the V20 Gy, and the V30 Gy.

## Results:

The mean volume of the spleen was 165.6 cc (59.6–484.8 cc). For left lower lobe tumors, the mean Dmean and Dmax received by the spleen were respectively 5.34 Gy (0.37–28.75 Gy) and 19 Gy (0.64–67.7 Gy). For right lower lobe tumors, the mean Dmean and Dmax received by the spleen were respectively 0.49 Gy (0.06–0.8 Gy) and 1.30 Gy (0.17–2.23 Gy). The mean V20 Gy and V30 Gy were respectively 4.5 cc and 3.71 cc. Lymphopenia was noted after the end of treatment in 60% of cases (*n* = 9/15). This lymphopenia was of grade 2 in 78% of cases. The delta lymph calculated was correlated with the Dmean and the V30 Gy of the spleen with Pearson coefficients of 0.57 (*P* = 0.02) and 0.53 (*P* = 0.03), respectively. No correlation was noted between the other dosimetric variables analyzed and the risk of lymphopenia.

## Conclusions:

Our results showed a significant correlation between the Dmean and the V30 Gy of the spleen and the risk of lymphopenia during radiotherapy treatment for left lower lobe lung cancers. Given the impact of lymphopenia on survival, we suggest considering the spleen as an OAR with the following dose constraints: Dmean < 6 Gy and V30 Gy < 4 cc to limit the risk of lymphopenia. Prospective studies are, however, necessary to confirm these results.

# Abstract: 11 Efficiency and tolerance of trastuzumab emtansine in the treatment of HER2-positive breast cancer

**Published:** 2024-06

**Authors:** Asma Elj, Manel Keskes, Imen Taktak

**Affiliations:** Specific Medicines Unit, Northern District, CNAM, Tunis 1002, Tunisia

## Aim:

Trastuzumab emtansine (T-DM1) is an antibody-drug conjugate incorporating the human epidermal growth factor receptor 2 (HER2) targeted antitumor properties of trastuzumab with the cytotoxic activity of the microtubule-inhibitory agent DM1. It is indicated as monotherapy for the treatment of adult patients, with HER2-positive unresectable metastatic or locally advanced breast-cancer, who have previously received trastuzumab and a taxane separately or in combination.

## Methods:

We have included all breast-cancer patients who received their first T-DM1 treatment from the National Health Insurance Fund between 01/06/2021 and 30/06/2022.

## Results:

Our study included 18 patients with a mean age of 54 years. Cancer was initially non-metastatic in 10 cases of whom 8 were receiving adjuvant trastuzumab. One patient had non-metastatic cancer and was treated with T-DM1 postoperatively for an incomplete histological response. The first-line T-DM1 was prescribed for 2 patients who had metastatic relapse during adjuvant trastuzumab and second-line therapy in 6 patients after relapse or progression under trastuzumab and/or dual HER2 blockade. T-DM1 was prescribed for the remaining after at least one line of chemotherapy and trastuzumab and/or dual HER2 blockade. The average delivery time of the treatment by the polyclinic of the national social security fund was 47 days. The average number of cures received was 9. T-DM1 was well tolerated in all patients. Seven patients are still stable on T-DM1 with an average duration of stability of 11 months versus 11 patients who had progressed with an average time to progress of 9 months. Five patients died. Progression-free survival was 10 months and the overall survival was 3 years.

## Conclusions:

T-DM1 has already proven to be effective and safe in several studies. It appears as an effective treatment option in the treatment of HER2-positive breast cancer. This is a descriptive and retrospective study that does not require an ethical approval.

# Abstract: 12 Cytokines in nasopharyngeal carcinoma and prognostic correlations

**Published:** 2024-06

**Authors:** Khemissi Faten, Ayari Jihen, Zribi Aref, Lagha Awatef, Fehri Rania, Ghazouani Ezzedine, Haddaoui Abderazek

**Affiliations:** Medical Oncology Department, Military Hospital of Tunis, Tunis 1089, Tunisia

## Aim:

Nasopharyngeal carcinoma (NPC) is characterized by distinct geographical distribution and is particularly prevalent in East and Southeast Asia. Its incidence is increasing in Tunisian population. Chronic inflammation can promote tumor progression, invasion, and metastasis. This investigation aims to assess the serum concentrations of cytokines (IL-1, IL-6, IL-8, and TNF) and their associations with the prognosis of patients with NPC.

## Methods:

Serum samples were collected in a prospective cohort of 25 patients with NPC treated in the Department of Medical Oncology of Jendouba before treatment, 7 weeks, 3 months, and 6 months after treatment. The TNF-, IL-1 beta, IL-6, and IL-8 were measured by a solid-phase.

## Results:

The average age of our patients was 47.8 years (14–80 years) with a sex ratio of 2.57 (18 men and 7 women). We observed a correlation between the highest level of cytokines and the age group between 31–40. The study also found that the levels of IL-6 and IL-1 beta were higher in patients with stage T1-T2, while IL-8 was higher in patients with advanced stages. Our research demonstrated that by the seventh week following the end of treatment the cytokine levels had increased, these signs indicated a positive response to treatment and a favorable prognosis. Additionally, we observed a significant increase in the cytokines in metastatic patients; IL-8 (*P* = 0.01), IL-6 (*P* = 0.001), and TNF alpha (*P* = 0.038). These are often associated with disease spread.

## Conclusions:

TNF alpha, IL-1 beta, IL-6, and IL-8 have the potential to be considered as prognostic biomarkers in NPC. These biomarkers identify a reserved prognosis group of patients that require aggressive treatment and closer monitoring. Other clinical studies are needed to better understand the inflammatory profile, its correlation with the NPC, the particularity of Tunisian patients and to validate the prognostic value of these serum biomarkers. In addition, subsequent molecular biology studies would be possible to determine the secretory origin of these cytokines; the body’s immune cells or tumor cells. The study was approved by the committee for the protection of people of the Tunis Military Hospital, Decision number 73:55/2023/CLPP/Military Hospital of Tunis. Informed consent to participate in the study was obtained from all participants.

# Abstract: 13 A retrospective comparison between chemoradiotherapy and total neoadjuvant treatment in locally advanced rectal cancer: real world data

**Published:** 2024-06

**Authors:** Donia Dhib, Haifa Rachdi, Yosra Berrazaga, Myriam Saadi, Nouha Daoud, Nesrine Mejri, Hamouda Boussen

**Affiliations:** Medical Oncology Department, Abderrahmen Mami Hospital, Ariana 2080, Tunisia

## Aim:

Curative surgery after neoadjuvant treatment is the gold standard treatment for patients with locally advanced rectal cancer (LARC). Pathologic complete response (pCR) after resection has been described as an important prognostic factor of low rate of recurrence and a long-term overall survival. Neoadjuvant radiochemotherapy has long been the gold standard but in recent years total neoadjuvant therapy (TNT) is being adopted worldwide as a promising means to improve outcomes for patients with LARC. We aimed to study the clinical and pathological features and the treatment outcomes related to both strategies in a center of Tunisia.

## Methods:

This is a retrospective, monocentric observational study including patients diagnosed with LARC and treated in the Oncology Department of Abderrahmane Mami Hospital, Ariana, Tunisia between 2014 and 2022. Data regarding epidemiologic characteristics, diagnosis and staging, preoperative treatment received, surgical outcomes, including treatment response, and pathological stage were collected.

## Results:

We collected 61 patients, 47.5% were female and the mean age was 58 years old. The main cT stage was T3 with 70.5% of cases and 31% N2. Tumor location distribution was 1.6% upper, 52.5% medium, and 45.9% lower rectum. The 54.1% patients had good differentiation grade and 4 patients had signet ring cell carcinoma. We have 2 groups: 33 patients were treated with TNT with short course radiotherapy and 28 patients with neoadjuvant chemoradiotherapy. For the first group, all patients received short course radiotherapy which was well tolerated. Radiological response was seen in 79% of patients (48.5% complete, 30.5% partial). Twenty-four patients had radical surgery, with 66.6% achieving an R0 resection and 24.2% had a pCR. Two patients have progressed on chemotherapy with the appearance of bone metastases and both patients had signet ring cell carcinoma. Three sudden deaths after chemotherapy were reported in this group although there were no grade 3–4 chemotherapy toxicity reported and 1 postoperative death; respectively aged of 75, 70, 71 and 40 years old. For the second group, all patient received long course radiotherapy with concomitant chemotherapy; 25 capecitabine, 2 capox, and 1 folfox. Radiological evaluation with MRI was conducted for 22 patients showing 54.5% partial response, 31.8% stability and 13.7% locoregional progression. We reported one distant metastatic progression after chemoradiotherapy. Among 27 operated patients, 89.1% reached an R0 resection and one patient had complete pathological response. The tumor regression grade (TRG) after surgery is detailed as follows: 3 TRG1, 5 TRG2, 7 TRG3, 5 TRG4, 2 TRG5. No grade 3–4 toxicity nor deaths due to treatment were reported. Due to the short follow-up for patients in the first group, we were unable to compare relapse-free and overall survival.

## Conclusions:

This study results support the advantage of total neoadjuvant treatment to increase pPCR in LA-RC patients. The study is exempt from ethical approval. Written informed consent was obtained from the patient for publication of this case report and accompanying images.

# Abstract: 14 Prognostic value of EBV load in nasopharyngeal carcinoma

**Published:** 2024-06

**Authors:** Khemissi Faten, Ayari Jihen, Zribi Aref, Lagha Awatef, Fehri Rania, Ghazouani Ezzedine, Haddaoui Abderazek

**Affiliations:** Medical Oncology Department, Military Hospital of Tunis, Tunis 1089, Tunisia

## Aim:

Nasopharyngeal cancer (NPC) presents a real public health problem and its rate is gradually increasing in our Tunisian population. Infection with the Epstein-Barr virus (EBV) can promote tumor progression, invasion, and metastasis. This investigation aims to assess the correlation between the presence of EBV DNA and the prognosis of patients with NPC.

## Methods:

Serum samples were collected in a prospective cohort of 25 patients with NPC treated in the Department of Medical Oncology of Jendouba before treatment, 7 weeks, 3 months, and 6 months after treatment. The detection of EBV DNA was accomplished by real-time PCR.

## Results:

The average age of our patients was 47.8 years (14–80 years) with a male-to-female ratio of 2.57 (18 men and 7 women). The highest rate of EBV DNA was associated with the 41–50 age group. Seven weeks following the end of treatment, the viral DNA burden had decreased, which indicated a positive response to treatment and a favorable prognosis. Furthermore, by evaluating serum levels at three and six months after treatment, we observed a significant increase in EBV load in metastatic patients. This can lead us to conclude that the analysis of EBV load before and after treatment could be a prognostic and predictive factor of response to treatment.

## Conclusions:

EBV load have the potential to be considered as prognostic and a monitoring biomarker in NPC after treatment. The study was approved by the committee for the protection of people of the Tunis Military Hospital, Decision number 73:55/2023/CLPP/Military Hospital of Tunis. Informed consent to participate in the study was obtained from all participants.

# Abstract: 15 Prognostic value of lymphocyte infiltration in bladder cancer

**Published:** 2024-06

**Authors:** Fehri Rania, Ayari Jihen, Rejiba Rim, Mahjoub Najet, Khemissi Faten

**Affiliations:** 1 Department of Medical Oncology, Jendouba Medical Oncology and Radiotherapy Center, Tunisia; 2 Department of Medical Oncology, Military Hospital of Tunis, Tunis 1089, Tunisia

## Aim:

Bladder cancer is the most common malignant tumour in the urinary tract. It is a major public health problem. The aim of this work is to study the prognostic value of lymphocyte infiltration (TILs) in bladder cancer.

## Methods:

We investigated the prognostic impact of lymphocyte infiltration in urothelial carcinomas in a cohort of 30 patients treated in the Department of Medical Oncology of Jendouba over a period of 4 years. The quantification of TILs was performed on an hematoxylin-eosin (HE) slide most representative of the tumor and then analyzed in three groups TILs: low (less than 10% of immune cells in the stromal tissue of the tumor), intermediate (10–50%) and high (50%).

## Results:

Average age was 67 years (6–86 years). Median follow-up was 10 months. Overall survival (OS) was 9 months ± 1.4 months. Progression free survival (PFS) was 8.5 months ± 1.4 months. For patients with TILs > to 50% OS and PFS was significantly better compared to those with < to 50% rates. Moreover, there is no significant correlation between TILs and different clinical or histo-prognostic parameters.

## Conclusions:

TILs evaluation could be a new prognostic and predictive biomarker of treatment response in urothelial bladder carcinoma and particularly useful for identifying patients who may benefit from immunotherapy. Ethic committee of Military Hospital agreed.
